# “*Older people will die of old age. I’ll die of climate change”*: engaging children and young people in climate decision making for public health

**DOI:** 10.1186/s12889-024-19406-9

**Published:** 2024-07-12

**Authors:** Grace Arnot, Samantha Thomas, Hannah Pitt, Simone McCarthy, Elyse Warner

**Affiliations:** 1 Institute for Health Transformation, Faculty of Health, Deakin University, 1 Geringhap Street, Geelong, VIC 3220 Australia; 2 Curtin School of Population Health, Curtin University, Kent Street, Bentley, WA 6102 Australia

**Keywords:** Climate change, Climate crisis, Children, Decision making, Public health, Qualitative, Young people, Youth, Commercial determinants of health

## Abstract

**Background:**

The climate crisis is a significant risk to the health and wellbeing of children, young people, and future generations. While there are calls for children and young people’s engagement in climate decision making, current power structures limit their participation. This paper aimed to understand children’s perspectives about the impact of the climate crisis on their futures, their ability to influence climate decisions, and strategies and mechanisms to facilitate their greater engagement in decisions made about the climate crisis.

**Methods:**

Online in-depth interviews were conducted with *n* = 28 children (aged 12–16 years) across Australia. Photo elicitation techniques were used to prompt discussion about how the climate crisis impacted their futures, their ability to influence climate decisions, and strategies and mechanisms to engage them in climate decision making. A reflexive approach to thematic analysis was used to construct three themes from data. Images were analysed for ascribed meanings.

**Results:**

First, participants stated that they and future generations will inherit the climate crisis from older generations, specifically decision makers. Second, they described a need to address a range of age-related barriers that limit children and young people’s engagement in climate decision making, including perceptions about their capabilities. Finally, they discussed strategies and mechanisms to embed children and young people’s perspectives within climate decision making, including at civic and political levels.

**Conclusions:**

Children and young people have the right to be involved in decisions made about the climate crisis which significantly impact their futures, including their health and wellbeing. They argue for structural changes to embed their views in climate decision making, and describe a range of engagement strategies and mechanisms to structure their perspectives and knowledge with decision making processes. Furthermore, genuine involvement of children and young people in climate discussions must avoid youthwashing and tokenistic participation. The public health community can help address barriers to youth participation in climate action and should actively engage and collaborate with children and young people to facilitate their political and democratic influence over the climate crisis. This involves making room and creating an accessible seat at the decision making table to ensure their perspectives are embedded in climate decisions.

**Supplementary Information:**

The online version contains supplementary material available at 10.1186/s12889-024-19406-9.

## Background

The climate crisis has increasingly been recognised as a public health threat by health groups around the globe [1, 2]. The impacts of the climate crisis are not felt equitably [3], with children, young people and future generations set to bear the largest share of the climate burden and its impact on population health and wellbeing [4]. Anthropogenic climate change is an issue at the intersection of health and intergenerational justice. Failure to enact effective climate policy responses constitutes a violation of human rights, with the outcomes of climate inaction leading to a failure *“to prevent human rights harms caused by climate change*, *including foreseeable long-term harms”* [5, p. 2]. Researchers argue that the climate crisis specifically violates the rights of children, young people and future generations to a healthy and liveable future [6, 7].

The United Nations describe intergenerational justice as the idea that current adult generations, particularly decision makers, have a duty to safeguard the futures of children and young people [8]. In relation to the climate crisis, this means considering impacts such as the climate-related risks that older generations will allow younger and future generations to be exposed to, and the sustainable use of natural resources [8]. Ursin and colleagues [9, p. 2] highlight the injustice of this current power imbalance in adult driven responses to decisions that are made about the climate crisis, stating that *“no generation has superior claim to the earth’s resources*, *yet power is unfairly concentrated and accumulated among adult generations”.*

Individual climate actions are unlikely to produce the urgent and large-scale changes necessary to address the climate crisis and as such there is a critical need for *“systemic changes that will reduce everyone’s carbon footprint*, *whether or not they care”* [10]. A core tenet of redesigning foundational systems involves redistributing power across powerless and vulnerable groups [11]. This involves examining the systems and structures that have historically limited children and young people’s ability to contribute to policy decisions and practice democratic citizenship. Climate education and research initiatives for children and young people have tended to focus on promoting individual actions, for example limiting personal energy use [12]. A greater focus is needed on developing their capacity to address the complex relationship between climate and broader systemic factors such as power structures [13], and to be civically engaged in actions that produce sustainable structures and policies [14]. Further, approaches to engaging children and young people must adopt an intergenerational justice lens and consider the unique barriers and challenges that they face in seeking to influence decision making [15, 16].

There are increasing calls from public health experts for children and young people to be involved in decisions about their futures, particularly in relation to the climate crisis [17–19]. This includes recognising children and young people as valuable democratic citizens in their own right with unique perspectives and knowledge, rather than as future democratic actors as adults [20]. Human rights agreements such as the Convention on the Rights of the Child [21] support children and young people’s involvement in decision making spaces, and frameworks have been developed to facilitate their inclusion in policymaking [22, 23]. Children and young people themselves have proactively sought to exert power and agency over the climate crisis and their futures by engaging in acts of environmental citizenship, including participating in climate justice protests [24]. However, there remain limited opportunities for them to participate in the decisions that are made about the climate crisis. Furthermore, there is a need to develop strategies and mechanisms to engage them in decisions made about the climate crisis, as well as other issues that impact on their health and wellbeing [15, 19]. Children and young people should not be made to wait until they enter legal adulthood and leave school before they have the right and the ability to influence decision making. Rather, they should be considered legitimate political actors at their current age [19, 25].

This study aimed to understand how children (aged 12–16) view the impacts of the climate crisis on children, young people and future generations. The study considers what they think about their ability to influence climate decisions, and the range of strategies and mechanisms that could be used to engage them in climate decision making. The study was guided by three research questions:

1. How do children think that the climate crisis impacts them and future generations?
2. Who do children think has the power to act on the climate crisis?
3. How can children be engaged in decisions made about the climate crisis?

Given the impact of the climate crisis on public health [1, 2], and based on recommendations by children in this study, this article then discusses how the public health community can help engage children and young people in climate decision making and addressing existing systems and norms.

## Methods

### Approach

This paper was part of a broader qualitative study that investigated children’s perspectives about the impact of the climate crisis on their futures and their ability to influence climate decisions. Australian children (aged 12–16 years) were invited to take part in in-depth interviews using photo elicitation techniques.

This study was shaped by an experiential and interpretivist qualitative approach [26], which focuses on exploring and understanding *“participants’ subjective experiences and sense-making”* [27, p. 3]. These insights can be used by public health researchers to better understand lived experiences and how individuals are impacted by specific policies and decision making [28]. In relation to this study, this type of research is important to help understand the depth and nuance behind children’s diverse experiences, perspectives and knowledge in relation to complex discussions about climate [25, 29]. We also used a critical qualitative approach to inquiry which involves examining the systems and structures that contribute to inequities, and using these findings to generate recommendations to drive change [30]. This approach was consistent with the public health position of the research team, which recognises that decisions by powerful actors can influence and uphold unjust and unhealthy systems and influence public health policies [31]. This position also recognises the need to create systemic change to drive meaningful climate action [32], and the need for the public health community to more critically evaluate the impact of structural determinants of the climate crisis [33].

The study used photo elicitation techniques to help prompt children to discuss the impact of the climate crisis on their futures, their perceptions about their power to enact meaningful responses, and strategies and mechanisms to strengthen their own engagement in climate decision making. Photo elicitation is a technique (influenced by visual methodologies largely from sociology and anthropology) in which images are discussed and analysed for meanings and symbolic representations [34]. This allows participants greater opportunity to *“express the nature and meanings of their life worlds in more depth”* [35, p. 8]. Such creative approaches are increasingly being used in research with children [36] and young people [37], with the use of imagery and photos aiding socio-political development, and facilitating a collaborative approach to research where children and young people become producers of knowledge [38]. The study was conducted entirely online. Online studies have a range of benefits including providing a more comfortable environment for hesitant populations, particularly children who may feel more comfortable participating via technology [39], and a more cost effective method for recruiting a geographically diverse sample [40]. Challenges of online studies include technology access or internet connectivity, as well as the potential for difficulty in building rapport over video calls [41].

Ethical approval was received from the Deakin University Health Ethics Advisory Group (HEAG-H 159_2021).

### Sample and recruitment

Most research exploring children and young people’s perspectives about the climate crisis has involved older adolescents and young adults, including their views about climate disasters [42], resilience [43], and climate decision making [15, 25]. To explore the perspectives of younger adolescents and children, individuals aged 12–16 years across Australia were invited to participate. Relating to sample size, qualitative studies do not typically seek large samples that seek representativeness or generalisability. Rather, qualitative studies seek depth of ideas and richness of detail [44], with the sample size being guided not by specific procedures or methods, but by *“shared methodological principles for estimating an adequate number”* [45, p. 1754]. Using this approach, researchers seek to collect enough ‘information power’ to *“develop new knowledge”*, with Malterud and colleagues [45, p. 1759] noting that *“the more information the sample holds*, *relevant for the actual study*, *the lower number of participants is needed”.* Guided by these principles, the present study aimed to recruit *n* = 30 children which was considered appropriate to provide enough information to collect a range of views about the climate crisis, and to answer the study research questions.

To recruit children aged 12–16 years, parents were approached and were the main point of contact for the study. Two recruitment strategies were used. First, recruitment notices were posted across social media platforms (Facebook, Twitter, and Instagram), climate organisations (such as parent climate action groups), and local pages (such as neighbourhood and community groups). Second, to diversify the sample, a recruitment agency was used. The research team provided the agency with the study recruitment flyer and Plain Language Statement, which the agency emailed to parents/guardians who were signed up to the agency and had indicated that they had children interested in engaging in research opportunities. Parents/guardians were able to request further information and were encouraged to discuss the study with their child, before being connected with the research team by the agency via email. For both recruitment strategies, electronic written and verbal consent were obtained during two separate stages. First, prior to the interview, the parent/guardian was encouraged to share and discuss the study with their child, then the child and their parent/guardian were required to sign and return the consent form attached to the Plain Language Statement. Second, at the beginning of the interview, the researcher also explained the study in detail and took the child through the topics of discussion and key concepts. The researcher reminded the child that if further support was required following the interview, helpline support was available. Participants were also reminded that they could skip any questions during the interview or stop at any time, and were then invited to ask any questions about the study before the interview commenced. This multi-step process of obtaining consent aimed to ensure that the child was fully informed before consenting to participate. Taking time to explain the study to children and to answer questions was important given that Wild and colleagues [46, p. 1] note that children recognise *“data as power”*, and want to know that their data is *“in safe hands”*, as well as the intended purpose behind the use of their data.

Twenty eight participants were included in the final sample. Thirteen participants were recruited via recruitment notices and fifteen via the research recruitment agency.

### Data collection

Data collection occurred from March – November 2022. Once children had been recruited and consented to participate in the study, they were asked to collect four images relating to the impacts of the climate crisis and the ability to influence climate decisions. Children could take their own photos (making sure to protect the confidentiality of individuals in their photos) or gather images (or visual materials, e.g., videos, memes, news headlines) from media sources such as newspapers and online spaces. Gathering images online was intended to foster an engaging and accessible research opportunity for children, particularly given COVID-19 restrictions at the time of the study.

Children then completed a one-hour, semi-structured, one-on-one interview with author one via the video conferencing platform Zoom, which recorded and auto-transcribed the interview. An interview schedule was employed to guide discussion which began with socio-demographic questions about age, gender, school level, town and state of residence, and some discussion about the type of area they lived in, such as if it was more metropolitan or regional. The main interview then began. The interview prompts discussed in relation to this paper included: (1) the impact of the climate crisis; (2) the impact on the futures of children, young people and future generations; (3) their perspectives about their power to meaningfully influence climate action, and challenges they faced in engaging in climate action; and (4) ways in which they would like to be engaged in decisions made about the climate crisis. Children responded to each prompt by sharing their perspectives and knowledge, with the researcher prompting for greater detail or meaning where the conversation focused on pertinent or novel topics, or where the child demonstrated particular enthusiasm for a topic. At the introduction of each of the four prompts, children were invited to share and discuss their respective images in relation to each of the prompts and follow up questions from the researcher. Some children shared their own screen via Zoom when showing their images while others, if they felt more comfortable, sent their images through to GA prior to the interview so that she could share their images for the child. GA alternated with the child sharing their own videos and images at different stages of the interview, prompting for their perspectives about mechanisms such as youth climate justice protests and other youth climate advocacy initiatives. Using these prompts, children discussed how they viewed their own ability to impact decisions made about the climate crisis, and how they would like to be more engaged and have greater influence. This back-and-forth sharing of visual materials, in combination with prompts and the participants’ own ideas, helped to foster a more youth-led approach. In the pursuit of *“avoiding ‘staged and superficial’ additions of youth participants into projects”*, it is crucial that researchers are continuously reflecting on ways to engaging younger populations as co-researchers [47, p. 11].

### Data analysis

A reflexive approach to thematic analysis (RTA) [48] was used to analyse interview data. Analysis was an ongoing process throughout both data collection and the formal analysis stage. The use of RTA involves constantly reflecting on and challenging researcher assumptions about the data [49]. The research team met regularly to discuss any perceived power imbalances and reflect on ways to address these [50]. For example, GA reflected on specific interactions with children during the interview and reported these to the broader research team for feedback about how to navigate or respond to similar interaction in the future. Other times, GA reported back key ideas (including those with particular relevance to the study aims, or that were novel and had potential for greater exploration) that had been raised by participants, which the research team was then able to reflect on and integrate into the survey questions for future interviews to gain a greater understanding of that idea. Once all interviews were completed, the six-phase process of RTA was applied (described in Supplementary File 1). Meanings and symbolic representations ascribed to images by children during the interview were used to illustrate key ideas in the data (for example, sharing an image of the Earth in a half-healthy and half-damaged state to represent their perspectives about the impact of the climate crisis on their futures).

## Results

The characteristics of the sample are outlined in Table 1. A total of *n* = 28 children participated. They were aged between 12 and 16 years (µ = 13.8 years), with a median school level of Year Eight (*n* = 6, 21.4%). Just over half of participants identified as male (*n* = 16, 57.1%), and most were located in the two most populous Australian states of Victoria (*n* = 11, 39.3%) and New South Wales (*n* = 10, 35.7%). Four out of five participants lived in a metropolitan area (*n* = 22, 78.6%).

**Table 1:** General characteristics of *n* = 28 Australian children (aged 12–16 years)

Characteristic	*n* = 28	%
**School year** Year 6 Year 7 Year 8 Year 9 Year 10	2 7 6 7 6	7.2% 25.0% 21.4% 25.0% 21.4%
**State** Victoria New South Wales Queensland South Australia Tasmania	11 10 5 1 1	39.3% 35.7% 17.8% 3.6% 3.6%
**Type of area** Metropolitan Regional	22 6	78.6% 21.4%

Three themes were constructed from the data. These are outlined in Table 2.

**Table 2:** Themes constructed from *n* = 28 Australian children’s (12–16 years) views about their engagement in climate decisions

Theme	Sub-theme
**Theme One: Children**,** young people and future generations will inherit the climate crisis.** Because of the climate crisis, participants perceived that children, young people and future generations would not get to enjoy the “natural beauty” of the planet, nor a healthy and liveable future. They largely identified decision makers from older generations as responsible for creating and passing on the climate crisis.	• The climate crisis threatens the health and quality of life of current and future generations (particularly in contrast to older generations). • The climate crisis threatens the enjoyment of the planet of current and future generations (particularly in contrast to older generations). • A range of reactions and emotions to the threat of the climate crisis. Some feel the need to enjoy the planet as it is now as it will only get worse. • Responsibility for the climate crisis lies largely with older generations, specifically governments.
**Theme Two: The need for structural change to overcome barriers limiting children and young people’s engagement in climate decisions.** Children in this study wanted to participate in decisions made about the climate crisis but were prevented by barriers and challenges associated with age. They felt their role in addressing the climate crisis was unclear, but were determined to continue acting on climate however they could.	• Climate inaction goes against understandings of how to treat others and responsible use of power. • Because current powerful actors aren’t acting in children and young people’s best interest, they want to influence climate decisions. • However, children and young people are limited to initiatives that lack power to directly impact climate decisions. • Other age-related barriers include school commitments, location, and public prejudice. • Children and young people receive conflicting messages about their role in the climate crisis but are resolved to drive the movement.
**Theme Three: Strategies and mechanisms to engage children and young people in climate decisions.** Participants perceived that there was a need for governments to meaningfully communicate and collaborate with children and young people about their perspectives on climate decision making. Children in this study described a range of engagement strategies and mechanisms to achieve this.	• The importance of listening and authentic engagement. • Engagement strategies – political and democratic. • Engagement strategies – intermediary and community building mechanisms. • School education about structural factors. • The need for strategies and mechanisms to consider varied capabilities and knowledge. • Must prioritise inclusivity and diversity.

### Theme One: Children, young people and future generations will inherit the climate crisis

Participants in this study described how children, young people and future generations will inherit the climate crisis, and a planet which will soon reach a *“point of no return”* and become *“destroyed”.* They contrasted their expectations of their health and quality of life in their climate-affected futures with the perceived higher quality of life afforded to older generations. Participants described how older generations had grown up free from the stress of the climate crisis impacting their futures, as it was either not *“common knowledge”* or talked about until more recently, or that tangible signs of the climate crisis were less evident and/or had a lower impact. They also described how their parents and grandparents had been able to experience and enjoy a healthy and liveable planet, including being less impacted by extreme weather events or the effects of pollution more broadly, including air quality and associated respiratory issues such as asthma.

*When our parents grew up*, *just generally the place where we lived was a lot cleaner and less polluted as a whole*, *and now it’s like filled with smoke and stuff and they didn’t have to worry about things like is as much as we do now*. – 14 year old female, Queensland.

Participants stated that they had the right to the same experiences as older generations, including enjoying Earth’s *“natural beauty”*, such as *“beautiful habitats and forests”*, *“lovely beach*[es]*”* and *“the Great Barrier Reef*, *one of the seven wonders of the world”*. Many also considered the impacts on future generations and their loss of access to such enjoyment, including their own children.

*My grandma and grandpa*, *when they were growing up*, *they had like these beautiful places to experience and visit and things*, *and we might not have that*, *or my kids.* – 13 year old female, Victoria.

Some participants dealt with this fact through humour. This included bringing memes about melting ice caps and the need to eat less meat, as well as laughing while stating that *“we’ll have to move planets or something!”* to deflect the seriousness of the crisis facing them and future generations. However, when asked explicitly how they felt about not having control over the outcomes of the climate crisis, all participants stated that they were some combination of *“anxious”*, *“angry”*, *“frustrated”*, *“sad”* and *“upset”* about the injustice being done to themselves and future generations. They described how they felt like they had to appreciate their current quality of life and *“enjoy what I have”* because of the worsening crisis and predicted consequences.

*It’s taught me to really live in what I have now*, *because it might not exist in 20 years… seeing a clean beach and going*, *“man*, *I really better enjoy this now before it gets absolutely fucked”.* – 16 year old male, Queensland.

Some participants initially attributed responsibility for the climate crisis broadly to older generations and *“mostly older people”* who would not have to face the worst of the consequences of their decisions that drove the climate crisis. However, when prompted further, they identified the main perpetrators of the climate crisis as being governments and those who can *“make really big sweeping changes”.* Participants described their perception of decision makers as *“old white men”* and *“dinosaurs”*, highlighted by their images including the incumbent United States President Joe Biden at COP26, and a cartoon of suited, mostly white men in conversation. They described decision makers as having had, and continuing to have, the power to implement comprehensive policy responses including regulation, legislation, and a just energy transition, noting how *“they make the policies. They set the rules”.* However, participants described their perception that decision makers were unlikely to act to limit the fossil fuel industry’s activities given their vested interests and being *“money driven and money focused”*. Participants described the injustice of children, young people and future generations growing and living to see the worst of the climate crisis, while current older decision makers and powerful actors will *“die before anything really happens”*.

*Older people will die of old age. I’ll die of climate change… I feel like the younger generation actually needs to think about it because they’re gonna be living in that time and like*, *it’s gonna get worse and worse*, *and they need to do something about it*. – 15 year old female, New South Wales.

### Theme Two: The need for structural change to overcome barriers limiting children and young people’s engagement in climate decisions

The lack of action was *“astonishing”* to some participants, who expressed a general expectation that the government should act in the best interest of society. They questioned why decision makers and people with power weren’t making decisions that protected children and young people’s futures, or at least protected their own children. The lack of humanity associated with a lack of climate action could not be reconciled with participants’ own sense of altruism and how they understood their responsibility to their fellow humans, particularly as an *“elected official”*.

*I don’t think* [decision makers] *have to listen. I don’t think they have to do exactly what the protesters say. I just believe that they should. They should paint a picture in their minds showing that people need something. Like*, *if someone asks for something*, *you’d usually give it to them. It’s kind of like that*, *but it’s more than one person asking the government for their protection and for their safety from these extreme conditions.* − 13 year old male, Queensland.

Because of the failure by governments and industry to make decisions that addressed the climate crisis and prevent the associated harmful consequences, participants described how they wanted children and young people to have input into the decisions made about the climate crisis. They stated that there was a need to *“bring some younger voices in”* to inform decisions with their perspectives.

*We’re always being told what to do by the politicians and stuff and we’re trying to change that… It’s like we’re a little bit powerless and that there is someone more powerful who is still making those decisions for us*, *when really what we want is the power to inform those decisions.* – 14 year old female, Tasmania.

However, they perceived that their own power to engage in effective climate responses was limited by their lack of structural power. They stated that their young age presented a significant barrier to their participation in climate decision making, as well as the democratic engagement processes for citizens to engage politically, including voting and entering politics *“to make a difference”*. Participants described how, given their lack of access to these political and democratic avenues, children and young people around the globe were using grassroots advocacy and action to raise awareness about the urgency and seriousness of the climate crisis. These included climate justice protests, completing surveys, circulating petitions, and engaging in social media advocacy. These initiatives were perceived as valuable for community building, fostering hope and raising awareness, but not effective for shaping government and industry decisions.

*I don’t think* [children and young people] *have much* [power] *because they’re not*, *you know*, *like official powers and all that. But I guess we’ll try and protest and all that. They just don’t have any power as*, *like adults*, *I suppose.* – 15 year old male, New South Wales.

Participants described other age-related barriers that prevented their engagement. These included time commitments, particularly being busy with school, and not being of an age to make the choice to live in active metropolitan areas such as *“in a bigger city where they have lots of protests”.* They also discussed the impact of societal norms and expectations about children and young people’s ability to engage in decision making about serious issues. Participants stated that the public tended to see children and young people as uninformed and incompetent, which impacted the credibility of their messages about the climate crisis.

*I think some people might listen*,* but because we’re more young*,* most people think that we would do stupid things to help instead of actual resolutions*. – 12 year old female, Queensland.

Participants also described how they received contradictory messages about their role as children in the climate crisis. While often framed as *“too young”* or *“kids who should be in school”*, they were also labelled as *“agents for change”* and felt pressured to take action to address the climate crisis. In light of these conflicting expectations, they were especially frustrated by the assumption that children and young people lacked the knowledge or maturity to engage in climate decision making. As such, they raised a call to action - *“then teach us! We want to know”.* Regardless of how they saw their role in addressing the climate crisis, participants demonstrated a strong resolve to continue advocating for strong policy action on the climate crisis to protect their futures.

*We need to change now whether or not people want to*,* because the longer we leave it the worse it’s going to get… I guess I’m doing what I can and I don’t know really what else to do.* – 14 year old female, Tasmania.

### Theme Three: Strategies and mechanisms to engage children and young people in climate decisions

Participants suggested a range of strategies and mechanisms to engage children and young people in decisions made about the climate crisis, and highlighted the importance of building their power and agency to shape decisions that impacted them. First, they called on decision makers to *“please just listen”* to *“us*”, *“the people”*, and *“young people”* about the urgent need for action on the climate crisis. Participants emphasised the need for genuine and authentic engagement. This included ongoing, regular, and open communication with decision makers, and the importance of supporting children and young people by listening to them and making them “*feel heard”.*

*It’s really hard to make people in power*,* like politicians and people*,* actually listen. And I think loads of young people find it really frustrating when these people don’t listen.* – 12 year old female, Victoria.

Strategies and mechanisms varied in terms of sophistication and level of engagement. Some stated that they would like to be involved at a high-level and work with decision makers, such as being *“in positions of being able to talk to politicians”.* They were highly interested in *“being in the room”* and engaging alongside policy makers as *“youth delegations”* and representatives. Some participants were more interested in engaging in a democratic group setting, for example, in forums and citizen assemblies. Some participants stated that they were unlikely to engage in this level of political engagement, and that they were *“not really interested in that kind of thing”*. These participants and several others suggested developing democratic engagement opportunities that enabled some children and young people to engage while allowing others the choice to remain unengaged, such as creating a non-compulsory vote for older adolescents.

*I think that we have a lot less power and I think that*,* especially since it’s our future*,* we should have a lot more power. I think that the voting age should be lowered to 16. But I think that it shouldn’t have to be compulsory for 16 years to 18 years.* – 12 year old female, Victoria.

Participants also described the need for intermediary mechanisms to communicate with governments on their behalf. This included completing surveys and petitions to share their views, and engaging with youth and climate organisations to converse with governments and represent children and young people’s ideas and perspectives. Some participants identified the current research they were participating in - *“things like this”* - as an ideal mechanism for engagement. Social media and online communication were also considered powerful tools for engagement by children and young people, as well as for sharing information about the climate crisis and climate justice activities, and for building communities organised around climate action. Participants described the importance of many individuals forming an influential community, particularly if they could sway figures such as celebrities to advocate to decision makers.

*We are all small voices and we can be all these small voices that get together… if we can get those bigger voices to influence those other bigger voices then it might work.* – 13 year old male, New South Wales.

Participants also discussed the importance of climate education in school for developing their abilities to effectively advocate and act on climate. They described wanting to learn about the structural factors that shape the climate crisis and what they could do to drive meaningful change, rather than learning about individual actions such as recycling, taking public transport, and eating less meat. A few participants highlighted the importance of starting climate education *“at a young age”*, describing how knowledge about effective climate advocacy and action could become more common knowledge if children were taught during their early years. Some participants also described school as a site from which engagement could be facilitated. For example, the Australian Prime Minister could actively *“reach out to schools”* to seek children and young people’s input, or youth representatives from each school could meet with the Prime Minister to communicate the student body’s expectations. There was also a general agreement that schools needed to be more proactive about facilitating conversations about the climate crisis.

*I think it’s also important to have schools talking about it and make it not so much just like something that you see*,* but a conversation*,* if that makes sense.* – 14 year old female, Queensland.

Despite many participants rejecting narratives about the inability of children and young people to engage in decisions made about their futures and the climate crisis, some were aware of how their age and lack of experience might impact their ability to participate. For example, a few participants admitted that they did not think they would be able to clearly communicate their thoughts about the climate crisis in a face to face setting. To ensure that all children and young people were able to participate in climate decisions, participants discussed the importance of engagement mechanisms being accessible. This included developing mechanisms that were appropriate for a range of levels of capabilities:

*I’ve been on a few Zoom calls* [with climate advocacy groups] *and there were some things I couldn’t even understand what they were saying because I didn’t know what the big words meant and things*,* so it depends what age you’re in.* – 13 year old female, Victoria.

Children in this study also highlighted the importance of inclusion and engaging *“people that come from a diverse background”*, particularly in relation to First Nations in Australia. Participants discussed this inclusion as though it were an obvious element of the overall strategy to address the climate crisis, highlighting the extensive and in-depth knowledge that Indigenous groups have in relation to living sustainably and managing the land. They also underscored the importance of developing engagement strategies and mechanisms that suited the needs of communities.

*I think it’s got to be kind of more personalised and individual*,* like actually having* [decision makers] *talk to communities and stuff. Because even when we look at activism*,* it’s still very much focused on privileged communities*,* even within a country. And like*,* what different communities need is different*,* and so having that really*,* like*,* small group thing in a safe space where people can actually speak their mind and say what they want to say is really powerful because then we can actually get solutions that work for us.***–** 14 year old female, Tasmania.

## Discussion

**Fig. 1:**
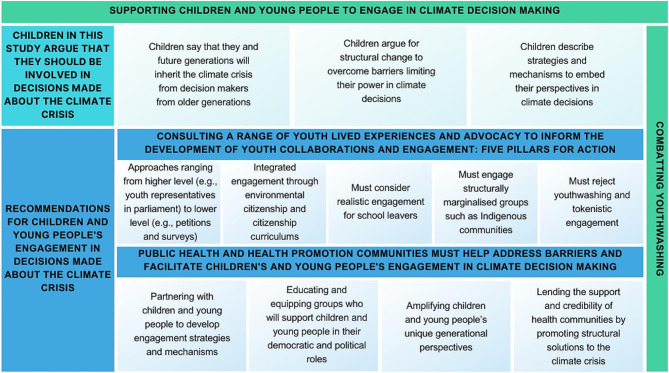
Supporting children and young people to engage in climate decision making

This study aimed to understand how children view the impact of the climate crisis on their futures, their own power and ability to influence climate decisions, and strategies and mechanisms to embed children and young people’s perspectives within climate decision making. This study also aimed to provide recommendations for the public health community for collaborating with children and young people to facilitate their engagement. An overview of the findings can be found in Fig. 1.

Children in this study, as well as children and young people in other studies [29, 51, 52], recognise that the climate crisis is driven by governments and the fossil fuel industry, and that the planet and their health and wellbeing is threatened as a consequence. Children described how, in comparison to older generations, including as recently as their own parents’ generation, today’s younger and future generations will experience a lower quality of life. This includes having fewer opportunities to enjoy the *“natural beauty”* that an unharmed climate and environment can offer. While they did not cite specific frameworks or acts that formally establish these rights (for example, the Convention on the Rights of the Child [21]), children in this study recognised that actors with the power to shape the climate were not making decisions that protected the right of children, young people and future generations to inherit a healthy and liveable planet.

The awareness of this intergenerational disparity was a key reason that children called for children and young people to be engaged in decisions about the climate crisis. They were angered by the unfairness of being subjected to the outcomes of climate-related decisions they themselves did not, and would not, make, and felt frustrated about their calls for action being ignored by decision makers. Rather than remaining passive victims of the decisions made by powerful actors, children in this study described the need to embed their perspectives into climate decisions. While there are some youth initiatives which facilitate meetings with decisions makers about policy issues that impact their lives [53–55], there are limited opportunities for meaningful involvement [15, 19, 56]. To ensure children and young people’s engagement is structured into climate decision making, there is a need to reimagine their democratic role, and the policymaking spaces that have historically been monopolised. Their political identities must be reconstructed to consider children and young people as not just future citizens, but present-day citizens with opinions and knowledge unique to their age group, and the right to democratic participation. This includes accessing legal mechanisms to hold decisions makers to account, such as in recent litigation against political actors across the globe [57–59].

Children described the need for engagement strategies and mechanisms to appeal and be accessible to a variety of abilities and interests among children and young people. In line with evidence about the necessity of diverse approaches to youth climate engagement [15, 20], they noted how children and young people may have varying levels of willingness or ability to engage. They also have different strengths and resources that enable their engagement [60]. For instance, some may want to be involved in high-level decision making, such as providing input into policies and meeting with decision makers such as the Prime Minister to discuss their perspectives and ideas. Others may be drawn to lower-level engagement opportunities such as completing surveys and petitions [15].

A more integrated and everyday way for children and young people to engage in climate action is through developing their skills as environmental citizens. ‘Environmental citizenship’ describes a citizen’s societal responsibility for maintaining a sustainable and healthy relationship between human activities and the natural environment [61]. Children and young people around the globe are already taking the initiative to practice environmental citizenship, such as school strikes and climate justice protests [24], and less visible initiatives including wildlife and biodiversity preservation [42]. However, there is a need for systemic changes that would structure environmental citizenship into everyday living and decision making to cultivate environmentally responsible practices among children and young people – one such way being the integration of environmental citizenship into school curriculums [62], and viewing schools as a site for democratic communication and development [63]. Children in this study called for climate education to start at a young age, including learning about the structural determinants of the climate crisis rather than just individual actions. Schild [64, p. 25] describes key indicators of a comprehensive environmental education, which include understanding, “*the physical*,* ecological*,* and social systems that interact to form environmental issues and problems”.* These outcomes link with cultivating agency, citizenship and democratic participation among children and young people.

Given that children and young people’s ideals and perspectives shift through their younger years, particularly after they leave school and gain lived experience [65], there is a need to understand which climate engagement approaches appeal to certain age groups. This includes the ways in which age and experience influence perceptions about the efficacy of strategies. For example, children and young people tend to be idealistic [66, 67], but as they age out of their younger idealism, they may be less interested in participating in initiatives such as climate justice protests. This is because street protests, while having value for community building and fostering hope, can be perceived to have limited value for driving climate policy change [15]. There is also a need for ‘climate empowerment’ to foster inclusivity and diversity by engaging children and young people at a population level [39], as well as structurally marginalised and underrepresented groups such as Indigenous nations [68, 69]. Further, inclusion of children and young people must reject tokenistic engagement [70], as well as ‘youthwashing’ [71]. ‘Youthwashing’ was coined to describe the insincerity of the fossil fuel industry’s engagement with young climate activists, and highlights the need to avoid inauthentic approaches and instead *“empower youth-led mechanisms*,* which are recognized and valued as such”* [72, p. 155]. Engagement strategies and mechanisms must promote genuine engagement, with children and young people considered current democratic citizens and political actors, rather than props to project an image of being youth focused.

There are a range of actions the public health community could take in engaging and collaborating with children and young people to develop strategies and mechanisms to embed their perspectives in decisions made about the climate crisis. First, there is a need to partner with children and young people to develop strategies and mechanisms to engage them in decisions made about the climate crisis [15, 19], including in addressing both political [25] and commercial determinants [29]. While there is a growing body of research that seeks to understand children and young people’s complex perspectives about the climate crisis [13, 25, 29, 73], there is a need for experiential research that involves engaging with children and young people for discussion about their diverse experiences, perspectives and knowledge. In this new capacity, children and young people should be supported to lead research rather than being passive participants who are called upon only when adult researchers have the ability and/or desire to engage them. To facilitate this shift, appealing and accessible strategies and mechanisms are needed to actively reach out to and engage children and young people, particularly younger children. For example, social media is a popular and familiar tool among younger populations for sharing climate information and messages across a wide audience and at a population-level [39]. Public health researchers might consider how social media could be used as a tool to involve younger populations in participatory approaches and engage them in a greater capacity as coresearchers [74].

Second, the public health community can promote children’s unique generational positionality in relation to the climate crisis, along with their perspectives and knowledge about the associated causes, consequences and solutions, which present a tangible and emotive voice to help situate the climate crisis as an act of intergenerational injustice. Doing so will require the public health community to strengthen their commitment towards genuine support and centring of children and young people in discussions about global health governance [75], including about the climate crisis [76]. Third, public health should identify and work with groups that are in a strong position to assist and support children in addressing the climate crisis, including researchers, decision makers, organisations, parents/guardians and schools [77]. Active engagement of these well positioned groups is important for informing strategies and mechanisms to support them in their role as stewards of children and young people’s climate education. Along with the public health community, these stakeholders must continue to question how to better engage children and young people in complex discussions and agenda setting, specifically about issues that impact their futures and their health and wellbeing. Finally, the public health community should also lend their own voice and credibility by advocating for structural change and solutions to the climate crisis [78].

### Limitations

There were two key limitations identified for this study. First, there were difficulties in recruiting a diverse group of children from all Australian states and territories, with most children living in the most populous states of Victoria and New South Wales. Future research may benefit from engaging a more geographically varied and dispersed group of children. This includes in Western Australia where the commercial and political contexts, including the presence of Australia’s mining industry, may influence children’s knowledge and perspectives. Second, informed consent was obtained using traditional approaches of written Plain Language Statements. These approaches may be difficult for children to engage with due to, for example, unfamiliarity with the concept of research, or being less interesting than more innovative approaches [79]. Researchers might utilise novel approaches such as creating an accessible and appealing short video to explain these studies and introduce concepts, including features such as being *“colourful*, *engaging and evidence-based”* [79, p. 6].

## Conclusions

Children, young people and future generations will inherit the climate crisis from older generations, yet decision makers and powerful actors continue to stall climate action. Children in this study were concerned about the consequences of the climate crisis on their futures, including impacts to their health and wellbeing. However, they recognised that they currently have limited structural power to influence climate decisions. Children and young people will be most impacted by the climate crisis, and they must be engaged in climate decisions to help drive urgent action by decision makers. This will involve fundamental changes to existing systems and structures, including acting upon children and young people’s right to have a say in decisions that impact their futures. The public health community should partner with children and young people to develop strategies and mechanisms to embed their perspectives in decisions made about the climate crisis. From a research perspective, strategies and mechanisms must be developed in collaboration with children and young people to engage them as co-researchers to ensure that their influence is embedded across all stages of the research process.

### Electronic supplementary material

Below is the link to the electronic supplementary material.

Supplementary Material 1

## Data Availability

The datasets generated and/or analysed during the current study are not publicly available as the individuals participating in this survey did not consent for their data to be shared beyond the research team but are available from the corresponding author on reasonable request.
