# Supplementary material for: “Older people will die of old age. I’ll die of climate change”: engaging children and young people in climate decision making for public health
Source: BMC Public Health. 2024 Jul 12;24:1869. doi: 10.1186/s12889-024-19406-9 (PMC11245781; doi:10.1186/s12889-024-19406-9)
Supplement: Supplementary file 1 — Supplementary Material 1 [file 12889_2024_19406_MOESM1_ESM.docx]

**Supplementary File 1**

**Applying Reflexive Thematic Analysis to interview data [Braun and Clarke, 2021]**

| **Step** | **Application** |
| --- | --- |
| 1. Familiarisation with the dataset | Following interview completion, Zoom generated an interview transcript. The transcript was revised by the interviewer whilst simultaneously listening the audio recording to correct transcription errors. Preliminary ideas for codes and themes were noted during this process, which were discussed and debated during meetings with the research team to help challenge assumptions about the data. |
| 1. Coding | The interviewing researcher and first author led the coding phase, with the research team regularly reviewing decisions made about the data analysis. Data was reviewed systematically for patterns and key concepts, first focusing on semantic and then latent ideas. The research questions and literature associated with this study were subject to active revision as data were continually analysed. |
| 1. Generating initial themes | Once a range of concepts and meanings had been constructed, they were categorised together to construct themes. For example, codes about climate inaction and intergenerational injustice were categorised under the theme C*hildren, young people and future generations will inherit the climate crisis***.** Codes were removed if they did not fit within the research aims and questions or themes constructed from the data. |
| 1. Developing and reviewing themes | Themes were reviewed to ensure that they were each a distinct part of the overall results narrative, and that they addressed the study’s research questions. |
| 1. Refining, defining and naming themes | The content and labels of each constructed theme were refined both before and during the results writing process. A short abstract was written for each theme and its subthemes (See Supplementary File Two). |
| 1. Writing up | During the writing of results, data were regularly revisited to ensure that the written results reflected the patterns, concepts and meanings within the data. Analytic and reflexive processes were also used in crafting the discussion and use of literature. |
